# Reports of Ohio Lunatic Asylum, Pennsylvania Hospital for the Insane, Friends’ Asylum, State Lunatic Hospital, Worcester, Mass., and Memorial of Miss D. L. Dix to the Legislature of Illinois

**Published:** 1847-04

**Authors:** 


### PART III.—BIBLIOGRAPHICAL NOTICES.

ARTICLE VI.

*Eighth Annual Report of the Directors and Superintendent of the Ohio Lunatic Asylum, to the forty-fifth General Assembly for the year* 1846.

*Report of the Pennsylvania Hospital for the Insane, for the year* 1846. By Thomas S. Kirkbride, M. D., Physician to the Institution. Philad. 1847.

*Twenty-ninth Annual Report on the state of the Asylum for the Relief of Persons deprived of the use of their Reason: to which is added an Account of the Asylum.* Published by direction of the Contributors. Third month, 1846.

*Fourteenth Annual Report of the Trustees of the State Lunatic Hospital, at Worcester, (Mass.,) Dec.*, 1846.

*Memorial of Miss D. L. Dix to the General Assembly of the State of Illinois, praying the establishment of a State Hospital for the Insane.* January 11th, 1847.

These documents evince an increasing interest in reference to the insane, alike gratifying to every philanthropist, and honorable to the age in which we live.

Success in the cure of patients seems abundantly to crown the operations of all these institutions.

From the first we make the following extract, which, as we have reason to know, is but an expression of public sentiment in our noble sister State:

"The directors are much gratified in being able to announce that this great enterprize of benevolence may now be considered as completed. *The work is done.* Its conception and execution belong to the people of the state of Ohio. The work is emphatically theirs. In all the agitations and mutations of parties—in all the monetary revulsions which have occurred since it was commenced, its progress has never been for a moment improperly delayed. Not a solitary voice of dissension has been heard upon the subject. Every year has witnessed the spectacle of a whole people, through their representatives, coming up as one man to lay their united offerings upon the shrine they had consecrated to the most touching and pitiable of human woes."

The subject is thus continued:

"It was remarked by the board, in their last annual report, that works like this, and the kindred charities which have grown up by its side are peculiarly the growth of christian times. Heathen lands have exhibited nothing similar. Antiquity had her wonders of science and of art—her Colussus—her pyramids—her triumphal arches—her amphitheatres and temples—which surpassed the proudest work of modern times—but in her palmiest days she had no Asylum for the insane—no educational institutions for the dumb and blind. The sepulchres of her history contain no trace of such a conception. Christianity was indispensable to their production. Between them and it, is the inseparable relation of cause and effect. How benign, and how wide spread and deep-rooted must be the influences which have produced such results!

"The subject suggests some further remarks, no less just and striking. Our own country is taking the lead in the establishment and promotion of these charities. The capability of man for self-government is a problem long since solved by our history. The diffusion of knowledge, the development of mind, and the highest prosperity are shown to be the fruits of free government. Its enemies still insisted that it must necessarily prove unfavorable to the progress of religion and benevolence. The rise and growth of these beautiful charities among us, are evidences upon that subject of the greatest weight. They prove that self-governed man is capable of the deepest sympathy for the afflictions of his brother man, and of the highest efforts to relieve them. He permits neither the sordid spirit of gain, nor any other selfish purpose, in his public councils or his individual action, to obliterate the better feelings of his nature. He seeks out the most helpless of the children of sorrow, and makes them the object of his care and bounty. The entire community recognizes it as a great and solemn public duty to provide for them and provision is made accordingly. Young as we are, no country is in advance of us in this work. This is a triumphant reply to the imputation alluded to."

The institution is calculated to accommodate three hundred and fifty patients. It had under care, at the date of the report, two hundred and eighty-three, and the number was rapidly increasing as apartments were fitted up for their reception. During the eight years of its successful operation, it has, at almost all times, been full to crowding with patients from within the limits of the State.

The expenses for the support of the institution, including over two thousand dollars for improvements, repairs, and furniture, payment of officers, attendants and hands for the year, was $22.946 07. The average number of patients in the institution during this time was two hundred and forty-four—making the cost for each patient, covering all expenses, about $94 00 per annum, or about $1 80 per week.

The second of these is a report from one of the finest institutions in the country. It is a branch of the old Pennsylvania Hospital, in Pine street above Eighth, Philadelphia, which was incorporated in 1752, and which, notwithstanding a yearly expense of many thousand dollars upon free patients, has, by rise in the value of its property, and the donation of benevolent individuals, become so wealthy as, six years ago, to erect this noble branch on a farm of over one hundred acres, two miles west of the city, at an expense of near three hundred thousand dollars, and since, to keep it in successful operation. During the past year extensive additions have been made to the institution, providing room for many additional patients, which will soon be finished and ready for their reception.

The past has been its most prosperous year, both in reference to the number of patients applying for relief, and in the success of treatment.

The average number of patients is one hundred and seventy-three, and the number of those discharged cured is eighty-nine.

The third is from one of the oldest institutions of the country, having been opened for the reception of patients in the year 1817, and was at that time far in advance of any thing of the kind in this country. It is under the exclusive care and management of the Society of Friends, by whom it was founded. The average number of patients in the asylum during the past year is 57½. There were received 26, and discharged or died, 34. Of those discharged there were 15 cured, 6 improved, 7 stationary, and 6 died.

We make the following extract from the account of the asylum by the attending physician, Dr. Evans:

* * * "The real state of the houses for the reception and treatment of the insane, in Great Britain, was first disclosed to the public, by the report of a committee of the House of Commons, published in 1816. Credulity itself is staggered at the recital of the before unheard of cruelty practised and misery endured, within the walls of most of those institutions, many of which the public had been accustomed to regard with pride, as monuments of their liberality and benevolence. There were, however, a few honorable exceptions, and conspicuous among these was the Retreat near York, which was projected by the Society of Friends as early as 1792, the same year in which Pinel commenced his celebrated reform in the Bicêtre at Paris. The plan of that Institution originated with a few individuals in the Society, who, having accidentally become acquainted with the manner in which the insane were habitually treated, resolved to rescue such of their fellow professors as suffered under that pre-eminent affliction, from the misery which surrounded them, and to place them in a situation where they would be subjected to a totally different course of management from that pursued in any of the existing establishments. Accordingly grounds were purchased, buildings erected, and in 1796 a considerable number of patients received, and a course of treatment carried, such as had never before been practised towards the insane, and which gave a rational ground to hope that their cure would be effected, or, at all events, their comfort and welfare secured. The Retreat was soon resorted to by others than Friends, and a short time the success obtained there demonstrated, beyond contradiction, the superior efficacy, both in respect of cure and security, of a mild and humane system of treatment in all cases of mental disorder. To the philanthropic members of that religious society who founded and conducted the Retreat, belongs (together with Pinel, who made some reformation in the horrible abuses of one of the Paris hospitals,) the credit, whatever it may be, of changing the course of treatment long pursued towards those deprived of the use of their reason, and restoring to them that sympathetic kindness and control which their affliction peculiarly demands. The example thus set was slow in extending its influence, as is evident from the state of the institutions throughout Great Britain, when the investigation before alluded to took place. That it had, however, a decided effect in awakening the public mind to the importance of a reformation in the insane hospitals, is shown by several parts of the evidence given before the committee of the House of Commons. Dr. Weir, Inspector of Naval Hospitals states, in his testimony, that 'the object of almost every insane institution, whether of a public or private description had been the *security* of those pitiable objects; comfort, medical and moral treatment being in a great measure overlooked; happily, however, for that class of society, the Quaker's Retreat at York, has at last convinced the world how much may be done towards the amelioration of their condition.' "

The fourth is also from one of the veteran institutions of this country. It is a State establishments, but has received one or two large legacies from private individuals.

The original plan and building was designed for the accommodation of one hundred and twenty patients, to which, during the fourteen years that have elapsed since it went into operation, have been added several additional wings, with detached buildings, so that, during the past year the average number of patients has been three hundred and fifty-nine, and the number who have enjoyed its benefits during the same period is six hundred and thirty-seven.

The per cent. of recoveries on all recent cases is seventy-nine; on old cases, that is on cases of more than one year's standing, it is twenty-eight; and on all cases, fifty-seven.

The following are notes taken of the post mortem examination of the remarkable and melancholy case of a girl who was, for a long time, deprived of almost all of her external senses, an interesting account of which was extracted from the thirteenth annual report of this institution, and published on page 68, Vol. I, No. 1, new series, of this Jourual:

" External appearance small, emaciated, pale.

" Dura mater not unusually adherent, nor were glandula pacchioni.

" Brain unusually firm; red points in section not more than usual; no unusual congestion; no unusual serum in arachnoid; perhaps one ounce of serum in the ventricles, and more at the base of the brain. On being removed from the cranium and turned over, the origin of the olfactory and the whole of the optic nerves were partly concealed by a considerable amount of false membrane, not recent; the nerves themselves were not softened and no pus or lymph was seen. Just at the forward part of the left middle lobe of the cerebrum, there was a greenish portion, two or three lines in superficial extent, under the pia mater, looking at first like a tuberculous mass. On cutting through this, there was seen a mass of disease, presenting two appearances, viz.: first, a white portion bespotted with red, the red consisting of minute coagulæ; second, surrounding this first part, there was a golden or yellow part which was somewhat different. The centre portion was an inch, more or less in irregular diameter; the other portion one half an inch and regularly defined—the substance of the brain outside of this being firm; this lay just in front of the optic thalamus, but did not reach it. There was another mass of disease precisely similar, just below the posterior cornu of the right lateral ventricle. This latter did not quite reach to the base of the brain, and was considerably larger.

"The cerebellum was healthy; no disease was seen in the first two or three inches of the medulla oblongata.

"There was universal adhesion of the pleura—abundant scattered tubercles in the lungs, but no considerable agglomeration.

"The liver was large, pale, and supposed, to be fatty.

"The spleen was of the usual size and healthy, except a portion of an inch in diameter, which was distinct, palish, and friable, looking like a commencing metastatic abscess.

"The peritonum exhibited, every where, a fine bright red vascularity, but was not sticky, and presented no pus nor recent lymph.

"The omentum was very much thickened, being in some parts one or two inches thick, and quite red on the surface. It cut like scirrhus, but had no tubercles in it. There was a portion of it reaching into the right iliac region, which was very large and thick. The whole peritoneal surface of the intestines was studded with little drops, looking at first like recent lymph, but they could not be scraped off with a knife.

"The uterus, fallopian tubes, and ovaries were one mass of tubercular disease."

The memorial of Miss Dix is a strong and urgent appeal to the legislature of Illinois in behalf of the insane of that State, which, we are happy to say, has not been made in vain. A bill was passed at its recent session, providing for a Hospital for the Insane, to be located at Jacksonville, and an especial tax was levied to raise the means for its erection.

After setting forth the necessity of the early treatment of insanity, by a host of authorities, and clearly showing that proper care and treatment can only be given in a hospital, she gives the following, with many other reasons, for complying with her petition:

"Of the urgent necessity for a hospital in Illinois, many are sensible who will read these pages; but there is, perhaps, a larger number to whose minds this claim presents itself under the view of no serious and positive obligation. A little inquiry will satisfy all who doubt, that this is either a great or an increasing evil. Illinois, according to the years since the country was settled, has a full proportion of insane, idiotic, and epileptic patients; not numerous enough merely to make it expedient to establish a hospital appropriate for their care and cure, for their own protection and the protection of others; but an uncompromising duty, from the voice of whose warnings and admonitions there is no mode of escape or evasion. Here humanity, receiving impulse from woe; selfish motives, claiming relief from anxiety and perplexity which never cease their distractions; and political economy, now more clamorous than ever, combine to hasten your efficient action upon this most solemn question. A few, the timid and superficial readers of their fellow men, but a few, will plead against appropriations for this work, on the unsound reasoning that their constituents will disapprove the measure; but I believe that it cannot be shown that the people at large ever manifest displeasure when their representatives appropriate their money to such objects as these. The citizens of Illinois, as other States, will not be found backward to make even some sacrifices, should these be required, when it is made evident that great sufferings exist within their borders, which they have the ability to mitigate, to control, and to limit.

"Legislators of Illinois! upon your action on this question rest the peace and happiness, the usefulness and the lives of thousands of your fellow citizens;—nay, your own immediate interests herein are indissolubly intertwined. Who shall say that the familiar friend, the revered parent, the child of his affections, the beloved wife of his bosom, aye, even he himself may not claim the guardian care now solemnly as urgently solicited for others? Timely provide for maladies which cannot be wholly averted, but whose dire distresses may be mitigated and oftener healed.

"Rise not from the grave and often perplexing deliberations, which claim your legislation, till you have added to acts bearing merely on the political condition of your State, this work of peremptory obligation to humanity. Retire not from these Halls in which honor, integrity, and justice should rule till you have rendered this noble service to your fellow citizens; a service which shall be commemorated long after you shall have passed from the active stage of this life; a service, the holy recollections of which will assist to smooth your path through the 'dark valley;' and which the recording angel shall inscribe in the book of life; 'For the memory of righteous acts shall never perish, neither in this world, nor in that which is to come!'" E.

